# Formulary Reconciliation: Implementation of a Comprehensive Approach to Formulary Maintenance and Standardization

**DOI:** 10.1177/00469580241271219

**Published:** 2024-08-12

**Authors:** Janine G. Martino, Craig Stevens, Brian Jung Hoon Park

**Affiliations:** 1 UC San Diego Health, Jacobs Medical Center, La Jolla, CA, USA; 2 UC San Diego Health, Hillcrest Medical Center, La Jolla, CA, USA

**Keywords:** formulary, reconciliation, hospital, pharmacy and therapeutics committee

## Abstract

At our institution UC San Diego Health, formulary qualifiers such as indication expansions and restrictions based on provider specialty, patient location, or patient characteristics are input as free text into an online formulary platform. Inconsistency in formulary categories and their descriptions since the implementation of the electronic system have led to confusion and inconsistent formulary application amongst staff. We reviewed 880 unique medications with formulary qualifiers to standardize both categories and language. There were 537 items with inpatient restrictions (eg, restricted to service), 147 items with a restriction to outpatient use only, 94 items with a formulation restriction, 91 items with associated guidelines, and 11 items with formulary expansions. Formulary status descriptions were updated to be consistent and clear. A standardized and well-maintained formulary, via formulary reconciliation, can provide concise and informative insight to the formulary status for frontline healthcare staff.

**What do we already know about this topic?**
Medication formularies are lists of medications approved for use within a particular hospital or healthcare system; they serve as a guide for appropriate medication use within that institution. While health systems are required to create and maintain medication formularies; the approach to and frequency of maintenance is not well defined.
**How does this manuscript contribute to the field?**
This manuscript provides a new and updated approach to formulary maintenance that we call “formulary reconciliation.” Formulary reconciliation describes an in-depth review of all medications and formulations; it is a comparison of formulary status and usage restrictions to current clinical practice, P&T committee discussions, recently published literature, medication availability, and costs.
**What are the manuscripts implications toward theory, practice, or policy?**
The manuscript may serve as an organizational guide to healthcare providers responsible for formulary maintenance and inform medication use policies.

## Introduction

Formulary management is an essential process that health systems use to ensure that efficacious, safe, and cost-effective therapy is delivered to patients. A health system formulary is a regularly revised compendium of medications that is reflective of current standards of practice and drives medication selection within that system. The Centers for Medicare and Medicaid Services (CMS) require that hospitals have a formulary system for reimbursement and accreditation purposes, ensuring that institutions can safely provide standard medications at reasonable costs.^1,2^ At a basic level, aside from CMS requirements, having an established formulary that is readily accessible provides an organized method for medical staff to be aware of which medications and formulations are available. While a well-maintained formulary attempts to provide the best clinical and economic outcomes, it is becoming increasingly challenging as there are over 20 000 prescription drugs currently approved by the Food and Drug Administration (FDA).^3^ Additionally, new approvals are increasing every year, many of which have complex clinical, financial, and operational implications.^4-6^ Certain health systems may face additional challenges if they tend to treat rare diseases, are disproportionate share hospitals, and/or have 340B program regulations to consider.

UC San Diego Health is a four hospital, 1101 bed health system with six outpatient infusion centers, a home infusion service, and numerous clinics which all utilize a single formulary. Our institution treats several special populations including bone marrow transplant (BMT), pediatric and adult burn, cystic fibrosis, pulmonary arterial hypertension, and hemophilia among others. The formulary policy states that “medications are generally added to the formulary for all FDA approved indications and acceptable uses listed in any CMS- approved compendium (AHFS-Drug Information^®^, Clinical Pharmacology^®^, Drugdex^®^, National Comprehensive Cancer Network^®^, Lexicomp^®^) unless the P&T Committee decides otherwise.” If a provider would like to regularly use a medication in an off-label capacity which is not listed as an “acceptable use” in one or more of the five CMS approved compendia, a formulary expansion must be presented to the P&T Committee. Conversely, if there is a desire to limit use to a certain patient population, approval by a certain provider specialty, or similar restriction, this verbiage must be presented to and approved by the P&T Committee. The outcome of P&T decision on that medication is then documented in an online formulary platform, FormChecker™ by Elsevier Inc., which houses the formulary information and displays this information in ClinicalKey^®^ (Elsevier Inc.). This platform allows for real-time updates, a display of available dosage forms and strengths, as well as drug monographs and guidelines. Within this product, the formulary status and its details, if applicable, are manually entered using a free text by the P&T coordinator or designee.

One of the challenges that our institution was facing was a lack of standardized formulary status language. For example, medications restricted by service line were described in several different ways, yet the intent was the same; “restricted to oncology” versus “prescribing restricted to hematology/oncology/BMT attending physician” versus “inpatient use is restricted to the oncology service only.” Some medications that were restricted to outpatient use only also included a service line or indication or both in the description, while others did not (ie, “ferumoxytol is restricted to CKD patients only in the outpatient setting”, and “ocrelizumab is restricted to neurology providers in the outpatient setting”). Lack of uniform descriptions and formulary categories has led to inconsistent documentation over an approximate 10-year period. This inconsistency has caused confusion for frontline Pharmacy Department staff and has at times led to inappropriate approvals or delays in care. For example, one case was noted in which treatment of immunotherapy related adverse effects was delayed by several hours to gain approval for use of infliximab; an indication that was deemed appropriate for inpatient use. The purpose of this project was to ensure the accuracy and appropriateness of formulary statuses, create a consistent language for formulary restrictions, and apply those rules going forward with new formulary additions.

## Methods

In this quality improvement initiative, a group of pharmacists comprised of the current P&T Coordinator, Medication Use Policy and Outcomes Specialist, and Bone Marrow Transplant/Immune Effector Cell Specialist convened in a series of meetings over the course of 12 months to review all medications with a listed formulary status, update the online formulary platform, and report changes to the P&T committee. A meeting lasting between 1 and 3 h occurring every 8 weeks was attended by all group members to work on this project. Each meeting built upon the progress of the one before; specific tasks undertaken during the meetings were conducting the data pull, reorganization of formulary categories, assessing and discussing the list of medications, determining if formulary statuses required an update, manually typing the clarified status into a spreadsheet, and entering updates into the formulary system.

General formulary categories included “Formulary,” “Non-formulary,” or “Restricted,” and a free text field allowed the communication of additional information such as clarifying the restriction. A report was generated and exported into a spreadsheet from the electronic formulary database that comprised the entire list of medications with formulary statuses contained within the FormChecker™ system. Notably, one active ingredient could represent multiple entries referred to as “line items” as each formulation or dose of a medication with a designated formulary status was listed individually. For example, oral metoprolol immediate release (25 and 50 mg), oral metoprolol extended release (25, 50, and 100 mg), and intravenous metoprolol each appeared as separate line items.

A list of pre-determined formulary status free text verbiage options, which we call a “qualifier,” was created in order to standardize and categorize the statuses. These included inpatient restriction, outpatient restriction, guideline restriction, and formulation (eg, extended release, immediate release, intravenous, etc.) restriction. Upon review, it was discovered that the pre-determined categories did not fully capture the variety of formulary statuses within the system. Due to the complexities of the P&T determinations and/or updates based on changes in practice, some of the entries fit into multiple categories. Prior to a line-by-line review, the categories were redefined by the group and standard language qualifiers were drafted. The category qualifiers were meant to be simple and concise to leave little room for misinterpretation or variety. Details of categories, their descriptions, and standardized language can be found in Table 1.

**Table 1.** Restriction Category and Qualifier Descriptions.

Restriction	Qualifier/standardized language
Inpatient restriction	• Restriction only in the inpatient setting, including but not limited to service line and/or indication
	• Standardized language: “Inpatient restriction: (eg, Hematology/Oncology)”
Outpatient restriction	• Medications allowed in outpatient setting, inpatient use is disallowed unless authorized
	• Standardized language: “Restricted to outpatient use only”
Formulation restriction	• Specific formulations that are restricted, which can also include a service line, outpatient, or indication restriction
	• Standardized language: “The following formulation is restricted to _________ (eg, service): _________ (eg, formulation)”
Formulary expansion	• An indication approved by the P&T committee that is not listed as an acceptable use in any of the Centers for Medicare and Medicaid Services (CMS) approved compendia
	• Standardized language: “Formulary expansion: ”
Guideline associated	• Use of the medication is restricted to what is prescribed by the institutional guideline
	• Text containing URL links were deleted and the institutional guideline was directly added as a direct hyperlink

Inpatient restriction denotes a restriction on use within the inpatient setting, which can include a restriction on which service lines can approve the ordering of the medication (eg, oncology, cardiology, etc.) and/or the indication for use. For example, “tocilizumab is restricted to the hematology/oncology services for the treatment of cytokine release syndrome (CRS).” Each restriction in this category was reviewed to confirm clinical appropriateness to ensure the restriction remains backed by evidence-based medicine for use in the corresponding indication or service line. When such a restriction came under scrutiny, the minutes of the P&T meeting at which the agent was approved were reviewed to ensure that the restriction wording captured the contemporary intent by the P&T Committee. The electronic health record (EHR) orders for restricted medications were also reviewed to check for “order questions” that the provider must answer to ensure that, if present, they aligned with the formulary restriction. Additionally, if the restriction was based on the high-cost nature of a medication in the past, the current cost was reviewed, and a restriction removal was proposed to the P&T Committee when appropriate. This process led to several formulary modification requests to align the formulary status with clinical practice.

Outpatient restriction signifies that outpatient use is allowed. Inpatient use of the same medication requires approval by an authorized pharmacist, pharmacy manager, or the P&T Chair via an internal process known as “inpatient request for an outpatient restricted medication” (IRORM). This distinction is important as the formulary spans all licensed treatment areas, both inpatient and outpatient. An example is obinutuzumab, which is restricted to outpatient use only and would require a request via the IRORM process for inpatient use.

Formulation restrictions within a specific medication can be used when there are multiple forms of the same medication, yet the indication, cost, or prescriber credentialing may impact its use. An example is the long-acting form of tacrolimus which is restricted to the Solid Organ Transplant and Hematology/Oncology services while immediate release formulation of tacrolimus is unrestricted.

Formulary expansion, or indication expansion, permits a P&T approved “off-label” use of a medication based on expert opinion and published peer-reviewed literature that is not captured as an acceptable use within one or more of the CMS approved compendia. All instances of formulary expansion were reviewed and approved by the appropriate P&T committee. One example is the approval of moxifloxacin for intracameral use, which is restricted to Ophthalmology.

Non-formulary denotes a medication that has not been previously considered for formulary inclusion or was considered and specifically voted as non-formulary. These medications, however, may still be ordered and dispensed following the P&T approved procedures. Firstly, pharmacists attempt to identify a formulary alternative and whether a pharmacist has P&T authority to adjust the medication order. In such cases, pharmacists will provide a substitution or contact the prescriber to order a substitute. If no alternative exists, the order may be processed as prescribed if clinically appropriate; in this scenario if the drug cost exceeds a certain amount ($5000) additional managerial approval is required.

Formulary clarifications were made throughout the process when an agent was identified as having a restriction that was found to be incorrect or after review of the historical P&T meeting minutes when the restriction required clarification. Several topical steroids that had never been evaluated by the P&T committee were mislabeled as “restricted” yet should have been listed as non-formulary. For drugs or drug classes associated with an institutional guideline, the guidelines themselves were reviewed to ensure they were current and available via a working hyperlink instead of listing the URL in the free text section. An example of a medication subjected to guideline restrictions is the use of andexanet alfa based on the institutional anticoagulation reversal guidelines.

## Results

**Figure 1.**
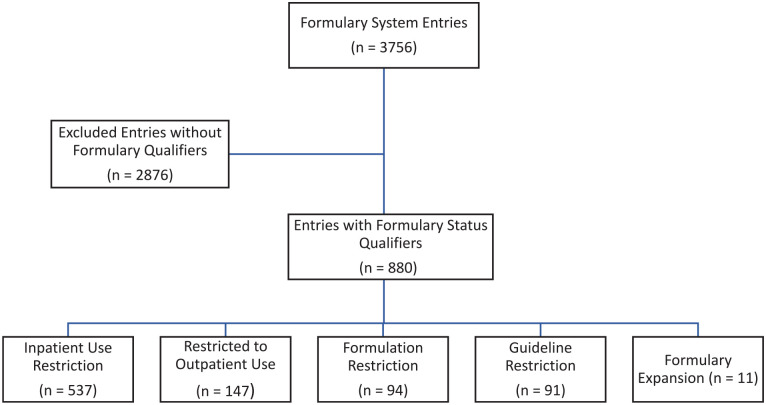
CONSORT flow diagram.

In total, 3756 line items appeared on the list of medications within the formulary system. After combining the doses and formulations, there were 1435 unique medications. As the intent of this initiative was to clarify and standardize formulary statuses, this list was further narrowed to 880 line items that contained free text formulary status qualifiers, which equated to 377 unique medications. Of the 880 line items, 537 contained restrictions on inpatient use, 147 were restricted to outpatient use, 94 had a formulation restriction, 91 had a free text guideline hyperlink, and 11 contained an expansion of indication (Figure 1 and Table 2). Additionally, 25 entries fell into multiple categories. Prior to program implementation, there were 309 distinct descriptions of formulary statuses. As an example, the following two descriptions were counted separately “At UC San Diego durvalumab is restricted to outpatient use only” and “At UC San Diego risankizumab is restricted to outpatient use only”; both changed to “restricted to outpatient use only.” After the reconciliation process, there were 155 distinct descriptions that were reorganized into 6 overarching formulary status descriptions. Most line items were easily incorporated into the new classification system based on the formulary status; recategorization was not common and only occurred after review of historical P&T meeting minutes for clarification or with P&T committee’s approval. All updates and clarifications were agreed upon unanimously by group members; if disagreement occurred within the group, pharmacists in other specialties or leadership positions were consulted to help inform the decision making. Any changes regarding the official formulary status of a medication were presented to and approved by the P&T committee. In one example exposed by this project, palonosetron was proposed to the P&T Committee to be changed from “outpatient restricted” to “inpatient restriction: hematology/oncology” based on current cost and usage data. An example of formulary removal was ritonavir/ombitasvir/paritaprevir, which was previously restricted to infectious disease service authorization but had since been removed from the market and was changed to “non-formulary.” Another more complex status update was changing nusinersen from “restricted for use in patients with spinal muscular atrophy who have insurance approval, prior to start of therapy. Only Neurology attending physicians privileged to provide intrathecal medication procedures may prescribe nusinersen” to “restricted to outpatient use only” which aligned with actual practice. Lastly, once the review was complete, a summary of the changes was presented to and approved by the P&T committee along with the updated formulary language. After approval, the P&T coordinator then manually updated the FormChecker™ system with all changes including updated guideline links. The entire process took about 12 months to complete.

**Table 2.** Number of Line Items Categorized by Formulary Status.

Formulary status category	
Inpatient restriction	537
Outpatient restriction	147
Formulation restriction	94
Guideline associated	91
Formulary expansion	11

## Discussion

Through this review, it was discovered that many formulary items with status qualifiers had inconsistent descriptions. Several formulary status descriptions were lengthy and confusing, while others were either no longer applicable or not followed in practice. The majority of the qualifiers were in relation to either inpatient restrictions or restricted to outpatient use only. The creation of simple and standardized formulary qualifiers will simplify staff training concerning formulary statuses and allow frontline staff members to easily understand and adhere to formulary decisions made by the P&T committee. Additionally, this standardization will allow the P&T coordinator to easily sort and filter to identify restrictions in the future. While we do use the EHR to provide decision support (eg, not listing non-formulary items on provider preference lists) we decided against utilizing EHR based alerts and order questions to manage formulary statuses to avoid alert fatigue and to provide a less labor-intensive method of housing and updating the statuses. Doing so would likely result in future inconsistencies between the multiple places where the information is stored. Lastly, while there is published literature on how to implement a formulary (eg, using automated systems), there is a lack of guidance on how a formulary should be maintained and continuously reviewed over the course of decades.^6-8^ Creating and utilizing a standardized formulary language can be the first steppingstone in this direction.

While the term “formulary maintenance” is used to describe the process of ensuring the accuracy and appropriateness of a formulary at predetermined intervals; perhaps a more appropriate term for this project would be “formulary reconciliation.” Aspects of formulary maintenance were already implemented at our institution but generally looked at a section of the formulary at a time either through medication use evaluations or drug class audits. While formulary maintenance is a standard practice in health systems, this approach mainly focuses on new drugs or drug classes as a whole and does not typically include a review of all medications in use within an institution. Formulary reconciliation goes a step further with creation of standardized categories and status descriptions, incorporation of a review of the historical P&T approval, current institutional uses (including off-label indications), current literature reviews, and up to date cost data. Without a wholistic approach that captures all items at once, years of a lack of standardization can lead to a wide variety of verbiages to describe what is intended to communicate the same concept.

Formulary reconciliation describes an in-depth review of all medications and formulations; it is a comparison of formulary status and usage restrictions to current clinical practice, P&T committee discussions, recently published literature, medication availability, and costs. While our project was limited to medications with formulary qualifiers, this process can be applied to all agents listed within a formulary. Additionally, this process can be expanded to review all the formulary medications within the electronic medical record to ensure that the formulary status and qualifier are consistent with our online formulary platform, although it would be ideal to use one platform to manage the formulary so the integration to electronic medical record system would be seamless. For the process to be successful, it is important to involve pharmacists with a thorough understanding of the institution’s formulary process, members of the P&T committee or sub-committees, informatics personnel, and those that also maintain a clinical practice. The updated formulary categories and descriptors are used by the current P&T Committee coordinators to guide formulary addition statuses ensuring they are placed into one of the newly approved categories and are added to the system in a consistent manner. While labor intensive, once set up appropriately, formulary reconciliation creates standards that should be followed for all future formulary decisions, theoretically requiring less time commitment when this process is repeated. We plan to repeat formulary reconciliation on an annual basis. As this process focuses mainly on reorganization and standardization, it can likely be replicated by any institution that maintains a “closed” or “restricted” formulary with an active P&T Committee.

## Conclusion

Formulary reconciliation is a useful tool to improve the efficiency of formulary management, and consistency in formulary statues helping to guide optimal medication use across a health system.
